# Classification of field wheat varieties based on a lightweight G-PPW-VGG11 model

**DOI:** 10.3389/fpls.2024.1375245

**Published:** 2024-05-14

**Authors:** Yu Pan, Xun Yu, Jihua Dong, Yonghang Zhao, Shuanming Li, Xiuliang Jin

**Affiliations:** ^1^ College of Mechanical and Electrical Engineering, Shihezi University, Shihezi, China; ^2^ Institute of Crop Sciences, Chinese Academy of Agricultural Sciences, Beijing, China; ^3^ Xinjiang Production and Construction Corps Key Laboratory of Modern Agricultural Machinery, Shihezi, China; ^4^ Engineering Research Center for Production Mechanization of Oasis Characteristic Cash Crop, Ministry of Education, Shihezi, China; ^5^ State Key Laboratory of Crop Gene Resources and Breeding, Chinese Academy of Agricultural Sciences, Beijing, China

**Keywords:** classification, lightweight, field environment, G-PPW-VGG11, partially mixed depth separable convolution, Android

## Abstract

**Introduction:**

In agriculture, especially wheat cultivation, farmers often use multi-variety planting strategies to reduce monoculture-related harvest risks. However, the subtle morphological differences among wheat varieties make accurate discrimination technically challenging. Traditional variety classification methods, reliant on expert knowledge, are inefficient for modern intelligent agricultural management. Numerous existing classification models are computationally complex, memory-intensive, and difficult to deploy on mobile devices effectively. This study introduces G-PPW-VGG11, an innovative lightweight convolutional neural network model, to address these issues.

**Methods:**

G-PPW-VGG11 ingeniously combines partial convolution (PConv) and partially mixed depthwise separable convolution (PMConv), reducing computational complexity and feature redundancy. Simultaneously, incorporating ECANet, an efficient channel attention mechanism, enables precise leaf information capture and effective background noise suppression. Additionally, G-PPW-VGG11 replaces traditional VGG11’s fully connected layers with two pointwise convolutional layers and a global average pooling layer, significantly reducing memory footprint and enhancing nonlinear expressiveness and training efficiency.

**Results:**

Rigorous testing showed G-PPW-VGG11's superior performance, with an impressive 93.52% classification accuracy and only 1.79MB memory usage. Compared to VGG11, G-PPW-VGG11 showed a 5.89% increase in accuracy, 35.44% faster inference, and a 99.64% reduction in memory usage. G-PPW-VGG11 also surpasses traditional lightweight networks in classification accuracy and inference speed. Notably, G-PPW-VGG11 was successfully deployed on Android and its performance evaluated in real-world settings. The results showed an 84.67% classification accuracy with an average time of 291.04ms per image.

**Discussion:**

This validates the model's feasibility for practical agricultural wheat variety classification, establishing a foundation for intelligent management. For future research, the trained model and complete dataset are made publicly available.

## 1 Introduction

With the rising global population, the need for high-yielding, quality wheat varieties is crucial amid shrinking farmland and climate change (Anagun et al., 2023). China, a leading wheat producer, contributed 41.54% to its total grain production in 2023 (Yearbook, 2023). In China, the family responsibility system governs agricultural development. This results in dispersed wheat cultivation without unified management, negatively impacting yield and quality (Dong et al., 2020). To reduce yield loss from depending on a single wheat variety, farmers often use diversified cropping strategies and cultivate multiple varieties. However, subtle morphological differences in leaves make visual identification of these varieties challenging, causing potential confusion in later field management stages. Accurate wheat identification using canopy leaf characteristics is thus a vital technique. This method assists farmers in distinguishing wheat varieties and guides the selection of those with superior yield and quality for future cultivation. Additionally, this technique offers researchers a way to monitor wheat growth and compare it with harvest data, contributing to the scientific basis for crop improvement and management strategies.

The traditional wheat classification depends largely on expert knowledge. However, this traditional method is inefficient and subjective (Ansari et al., 2021). Machine learning methods, known for their speed and convenience, are addressing these challenges in crop classification (Li et al., 2023a). Crop classification methods have advanced, with diverse approaches emerging (Alqahtani et al., 2023; Dogra et al., 2023; Li et al., 2023b). For instance, Dogan and Ozkan (2023) enhanced the Extreme Learning Machine (ELM) model using Harris Hawks’ Optimizer (HHO) and Particle Swarm Optimizer (PSO) for wheat grain classification. Results showed binary and multi-class accuracies of 99.32% and 95.95%, respectively. Singh et al. (2020) extracted wheat grain parameters using digital image processing (DIP), including groove area and asymmetry coefficient. They then classified wheat grains using a fuzzy clustering random forest (FCRF) based on the extracted parameters. This approach improved classification accuracy by an average of 7% over existing methods. Agarwal and Bachan (2023) preprocessed wheat grains by removing shadows and segmenting. They extracted color and texture features from the grains thereafter. Using cross-validation, they evaluated Support Vector Machine (SVM), K-Nearest Neighbor (KNN), Multilayer Perceptron (MLP), and Naive Bayes (NB) algorithms to select the optimal classifier. The classification accuracy reached 93%. Current wheat classification research, focusing on grain characteristics, is limited by growth stages and collection periods. Leaf image acquisition, possible throughout the wheat growth cycle, allows for continuous monitoring. Zhou et al. (2023) used a compact separation-based feature selection algorithm (FS-CS) to filter spectral and texture features from UAV images. They achieved over 70% accuracy in wheat phenology classification using a multilevel correlation vector machine (mRVM). While these studies report high accuracy, feature selection requires domain-specific knowledge and expertise.

The advancement of deep learning, especially the advent of convolutional neural networks (CNNs), has supported diverse image-based classification and recognition due to its robust feature extraction and task transferability (Yasar, 2023). Alom et al. (2023) successfully classified flowers, stems, and leaves of two oilseed rape varieties using transfer learning with five neural networks in visible light crop classification studies. Their method involving background removal and CLAHE preprocessing achieved 100% accuracy in flower classification and 97% in stem and leaf classification. Wei et al. (2022) trained on both fluorescence and white light images of five tea varieties using VGG16 and ResNet34. They observed that fluorescence imaging, induced by ultraviolet (UV) LEDs at 370 nm, yielded a higher classification accuracy of 97.5% compared to white light imaging. Gao et al. (2021) achieved up to 99.51% accuracy in classifying wheat leaves at three fertility stages using a bagging-based algorithm and ResNet models. However, their dataset was collected in a controlled lab setting with a uniform background. Sun et al. (2023) used MixNet XL CNN and KNN to classify 21 leaf types, achieving a 99.86% accuracy rate. Chen et al. (2021) introduced a localized soft-focus mechanism to MobileNet-V2, attaining an average accuracy of 99.71% for crop disease classification on the PlantVillage dataset. The core goal of the aforementioned research is to enhance model accuracy by increasing extracted feature values. However, deploying these models on hardware or mobile devices is challenging due to significant memory usage and processing power requirements.

Tang et al. (2022) introduced a geometric distance-based pruning and grafting (P&GGD) optimization strategy. This method successfully classified nine maturity levels across three tea types, balancing model accuracy and size. Consequently, the method achieved a classification accuracy of 96.296%, exceeding MobileNetV2 by 2.395% and reducing FLOPs by 45%. Yang et al. (2021) replaced VGG16’s F6 and F7 layers with Conv6 and global average pooling. They added a batch normalization layer, increasing peanut variety classification accuracy to 97.7%, an 8.9% improvement. Nasiri et al. (2021) modified VGG16, incorporating global average pooling, dense, batch normalization, and culling layers. Using leaf images in the visible spectrum (400-700 nm), this method classified six grape varieties with over 99% accuracy. In summary, these studies sought to develop lightweight models with reduced computational demands. This was achieved by integrating batch normalization and global average pooling, enhancing feature extraction. This strategy balances memory usage with classification accuracy effectively. However, these advanced strategies are seldom applied to wheat variety identification.

This study addresses the technical challenges in classifying and identifying wheat varieties by analyzing canopy leaf images from mobile devices in field settings for six distinct wheat types. The VGG11 model was chosen for its structural simplicity as our baseline, applying a series of targeted optimizations to improve its performance. The model was refined using three specific strategies.

First, we addressed feature redundancy and receptive field limitations in traditional convolution by replacing standard convolutions with partial and depthwise separable convolutions. This modification enhanced the model’s feature extraction and discrimination among wheat varieties.

Second, we replaced the fully connected layers with pointwise convolutions and global average pooling, significantly reducing the model’s parameters. This improvement reduces overfitting risk and maintains global connectivity, allowing the model to capture image information comprehensively and accurately.

Lastly, we incorporated an efficient channel attention mechanism to reduce background noise in the model’s predictions. This strategy further improved the model’s sensitivity to wheat features and its classification accuracy. Collectively, these modifications represent our novel approach to wheat variety classification and identification.

The enhanced G-PPW-VGG11 model was benchmarked against classical lightweight networks: FasterNet (Chen et al., 2023), MobilNet-V2 (Sandler et al., 2018), MobilNet-V3 (Howard et al., 2019), EfficientNet-V2 (Tan and Le, 2021), and ShuffleNet-V2 (Ma et al., 2018). The enhancements in this study improved wheat variety identification accuracy and achieved lightweight modeling. This lays a strong foundation for swiftly identifying wheat varieties in field conditions.

## 2 Materials and methods

**Figure 1:**
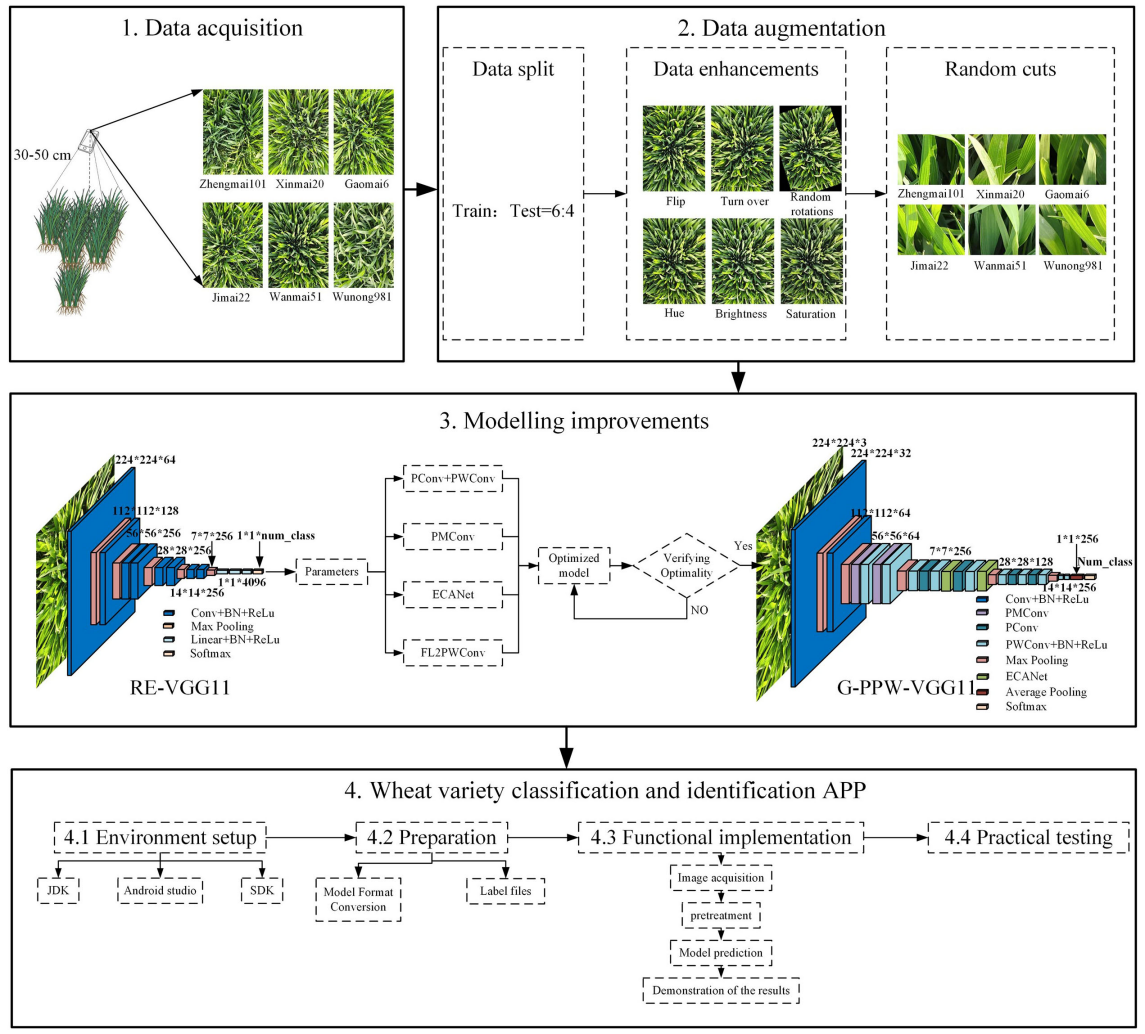
Flowchart of the experimental design.

The experimental design is illustrated in **Figure 1**. The following four subsections detail the data acquisition, preparation, modelling improvements, and wheat variety classification and identification application.

### 2.1 Data acquisition

**Figure 2:**
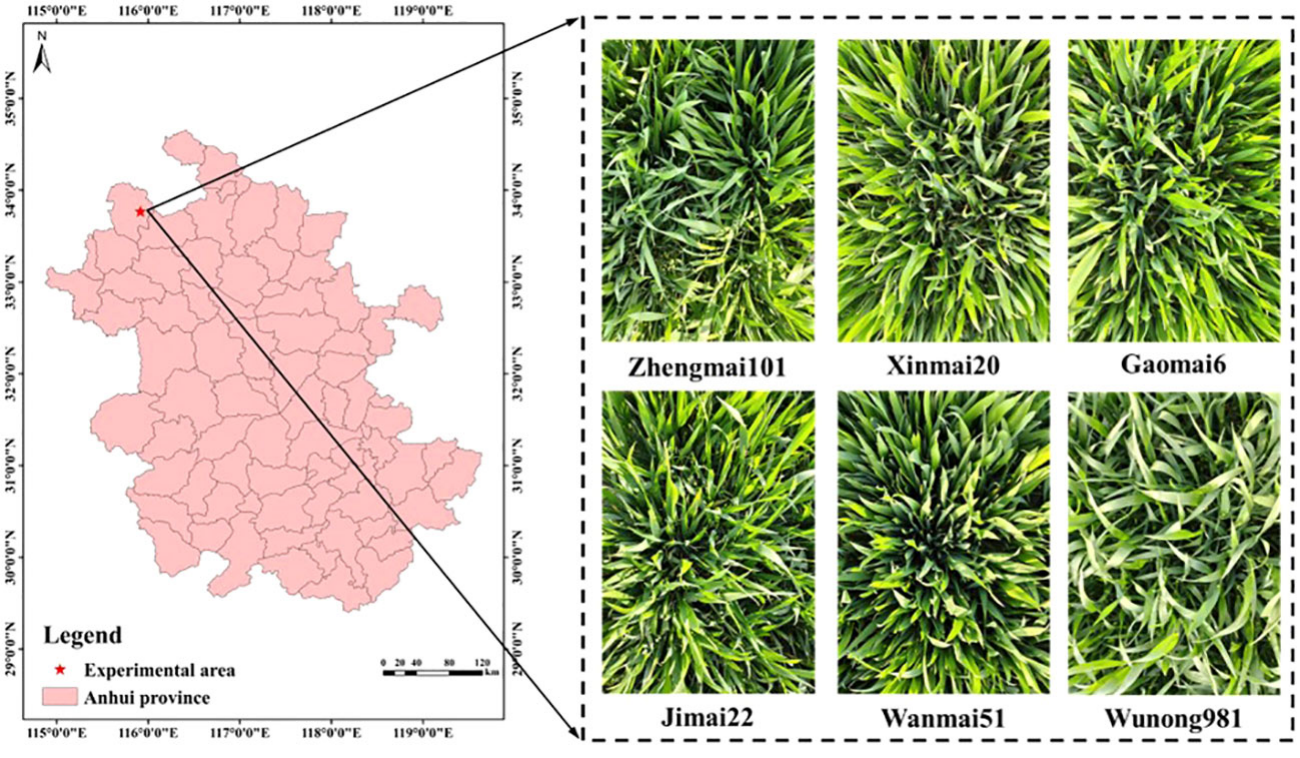
Experimental area (Bozhou City, Anhui Province) and data collected.

Experimental Area: **Figure 2** shows the location near County Road 015, Longyang Town, Qiaocheng District, Bozhou City, Anhui Province, China (33°46′9.06″N, 115°54′57.63″E), where the wheat dataset images were collected.

Collection Objects: This study includes six machine-seeded wheat varieties cultivated by local farmers: Zhengmai101, Xinmai20, Gaomai6, Jimai22, Wanmai51, and Wunong981.

Data Acquisition Equipment: Data was collected on April 04, 2022, from 14:00 to 18:00.

Data Acquisition Time: The data collection occurred on April 04, 2022, specifically between the hours of 14:00 and 18:00.

Acquisition Methods: The smartphone was held 30-50 cm above the wheat leaves, focusing on three rows with the leaves in the foreground and external elements as the background.

### 2.2 Data preparation

In this study, the dataset was randomly split into training (60%) and testing (40%) datasets. This split was designed to facilitate effective learning and improve the model’s adaptability across various classification scenarios. To enhance robustness, a subset of images was randomly selected for data augmentation, creating additional datasets that mimic natural conditions. The following augmentation techniques were employed:

1. Images were flipped vertically and horizontally and randomly rotated between -30° and 30° to simulate different angles and reduce positioning errors.
2. Brightness was randomly adjusted between 0.9 and 1.1 to mimic natural lighting variations.
3. Hue and saturation were independently adjusted between 0.9 and 1.1 to replicate the diversity of light and environmental conditions.

These augmentation strategies were designed to enhance the model’s generalization capabilities. After augmentation, images retained a resolution of 3,000×4,000 pixels. Using high-resolution images directly for training would significantly increase the number of parameters. Therefore, the enhanced images were first randomly cropped to 300×400 pixels, as shown in **Supplementary Figure 1**. These cropped images were then resized to 224×224 pixels. This process effectively expanded the dataset and reduced the model’s parameter count. **Table 1** outlines the detailed quantities of the processed dataset and the abbreviated names of the wheat varieties. The dataset can be accessed at the provided URL (https://pan.baidu.com/s/107ICGZOxmOXURkZQcHgbeQ) with the access code: 6666.

**Table 1:** Number of wheat datasets before and after processing.

Variety	Original image	Dataset		Data enhancement and cutting	
		Training	Testing	Training	Testing
Zhengmai101 (zm101)	157	94	63	1,963	1,054
Xinmai20 (xm20)	170	102	68	2,040	1,032
Gaomai6 (gm6)	153	92	61	1,902	956
Jimai22 (jm22)	159	95	64	2,213	1,087
Wanmai51 (wm51)	155	93	62	2,381	1,218
Wunong981 (wn981)	158	95	63	2,045	1,034

To assess the refined model’s stability, we constructed a cross-validation dataset using the original data. Ensuring data consistency as per **Table 1**, we employed a 3-fold cross-validation technique to evaluate the model’s robustness. **Table 2** provides a detailed overview of the dataset’s quantitative distribution.

**Table 2:** 3-fold cross-validation dataset.

Variety	Dataset 1	Dataset 2	Dataset 3
Zhengmai101 (zm101)	1,057	1,008	1,003
Xinmai20 (xm20)	1,049	1,089	1,105
Gaomai6 (gm6)	822	902	775
Jimai22 (jm22)	1,044	971	1,032
Wanmai51 (wm51)	986	920	948
Wunong981 (wn981)	1,071	1,064	1,077

### 2.3 APP development

#### 2.3.1 APP development environment

The application was developed on a Windows 10 operating system, utilizing a specific set of tools tailored for Android application development:

1. JDK (Java Development Kit) Version 17: This is essential for Java development, encompassing the Java runtime environment, tools, and basic libraries.
2. Android Studio Version 3.1: The chosen Integrated Development Environment (IDE) for facilitating app development and debugging.
3. Android SDK (Software Development Kit): Includes necessary tools, libraries, emulators, and documentation for Android app development, with a specific mention of the Android SDK Build-Tools version used.

#### 2.3.2 Functional design and implementation

The development process incorporates four optimization strategies into the mobile-optimized G-PPW-VGG11 model. Key steps include:

1. Image acquisition.

a) Local Photo Albums: “AlbumA.java” utilizes “android.intent.action.GET_CONTENT” to select images from the device’s album.

b) Camera Shooting: Establishing “Camera.java” for capturing images directly via the app, checking permissions with “ContextCompat.checkSelfPermission” and “ActivityCompat.requestPermissions”, and capturing images with “android.media.action.IMAGE_CAPTURE”.

2. Image preprocessing.

This method resizes images longer than 300 pixels to 300×300, then downscales them to 224×224 pixels to prevent distortion and preserve classification accuracy.

3. Application development.

a) Model Format Conversion: Converting the model from “.pth” to “.pt” format for Android compatibility and placing it in the “app/src/main/assets” directory.

b) Label File: Creating a “label.java” file for required label information.

c) Configuration File: Adding functional buttons for album and camera access, linking to “Albums.java” and “Camera.java” for respective functionalities.

d) APK File Generation: Outlining the process for generating an installable “.apk” file via the Android Studio environment.

## 3 Algorithm and improvement of wheat classification

### 3.1 VGG network model

Among various classification algorithms, the VGG network model is notable for its simplicity, making it an ideal basis for network optimization. The VGGNet family, proposed by Simonyan and Zisserman (2014), includes structures such as VGG11, VGG13, and VGG16. Aligned with the goal of devising a lightweight model for classifying wheat varieties, VGG11 was selected as the baseline due to its minimal layer count.

### 3.2 VGG11 network improvements

This study achieved model parameter optimization by adjusting the number of convolutional kernels in the VGG11 architecture. Considering the impact of convolutional kernels on model parameters, the original configuration was revised from 64, 128, 256, 256, 512, 512, 512, 512, 4,096, 4,096, classifier to a more efficient 32, 64, 64, 64, 128, 128, 256, 256, 1,024, 1,024, classifier. This modification streamlined the model without compromising classification performance.

Following Ioffe and Szegedy (2015), batch normalization (BN) was added after each convolutional layer. Integrating BN normalizes data distribution, enabling faster and more robust model training. The improved model, RE-VGG11, forms the basis for all further optimization experiments in this study.

Compared to lightweight models like MobileNet, EfficientNet, and ShuffleNet, RE-VGG11 faces several challenges:

1. Extracted feature values across channels show high similarity (Chen et al., 2023). Partial convolution was introduced to minimize feature redundancy.
2. The small 3×3 kernels in RE-VGG11 limit its receptive field’s diversity. Incorporating mixed depthwise separable convolutions alleviates this, reducing weight bias and enhancing feature integration from diverse receptive fields.
3. Fully connected layers comprised 92.02% of the original model’s parameters. Replacing them with pointwise convolution and global average pooling addressed this imbalance.
4. The field-acquired dataset contained multiple interference sources, prompting the introduction of an efficient channel attention mechanism. This enhances the model’s focus on relevant leaf information, improving feature discernment in noisy data and classification capabilities.

**Figure 3:**
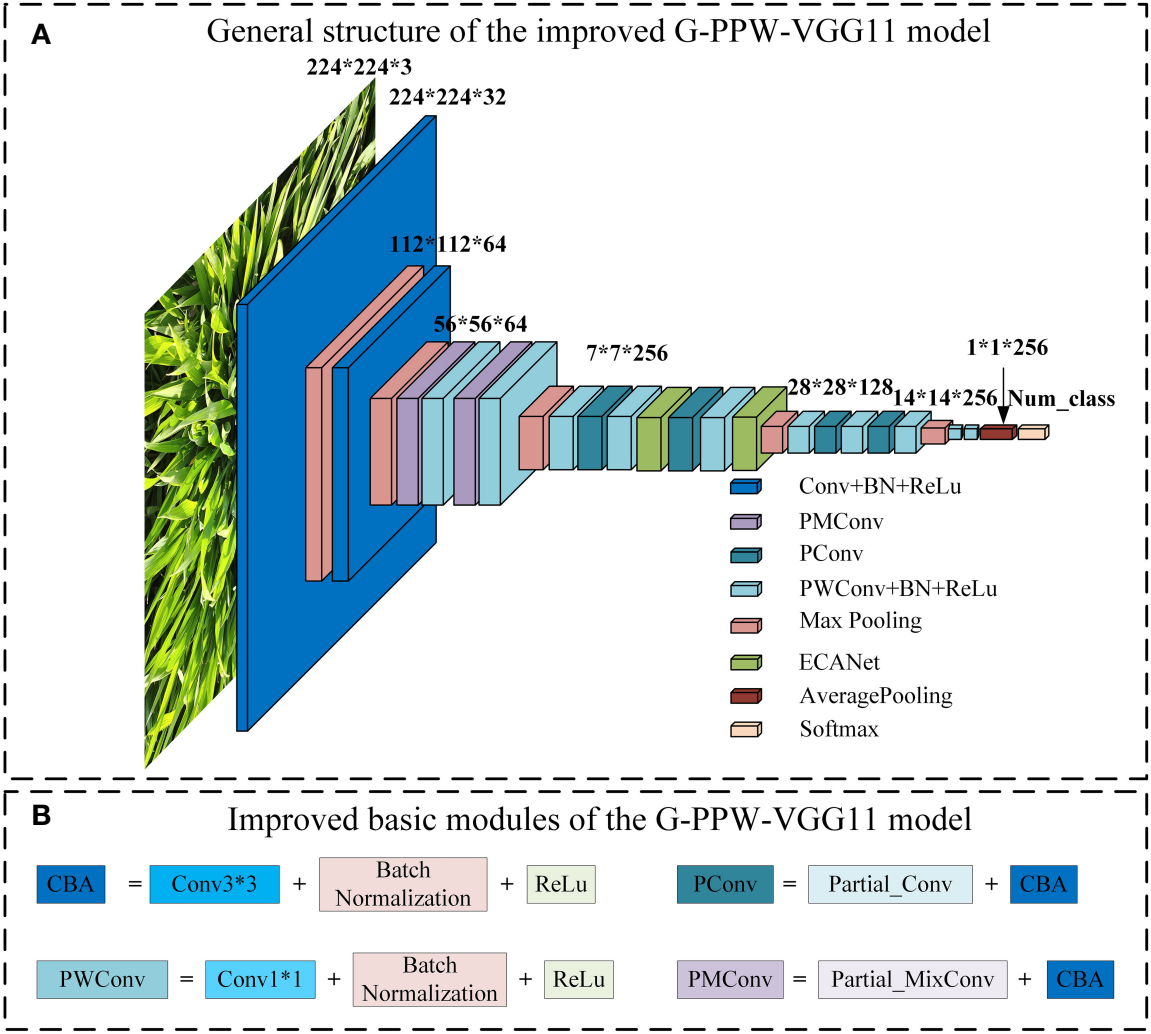
G-PPW-VGG11 model structure diagram **(A)** General structure of the improved G-PPW-VGG11 model. **(B)** Improved basic modules of the G-PPW-VGG11 model.

Enhancements addressing these challenges have been implemented in RE-VGG11. **Figure 3** shows the comprehensive structure of the refined model, G-PPW-VGG11, and its structural modules. Specific improvement strategies will be detailed accordingly.

#### 3.2.1 Partial convolution

The model exhibits a high degree of similarity among features extracted from different channels. This similarity causes the model to disproportionately favor redundant features during weight allocation, leading to excessive weighting (Chen et al., 2023). However, this bias in weight distribution leads to the neglect of smaller, yet crucial, feature components essential for accurate performance. The neglect of these components can undermine the model’s precision and, consequently, its overall performance.

**Figure 4:**
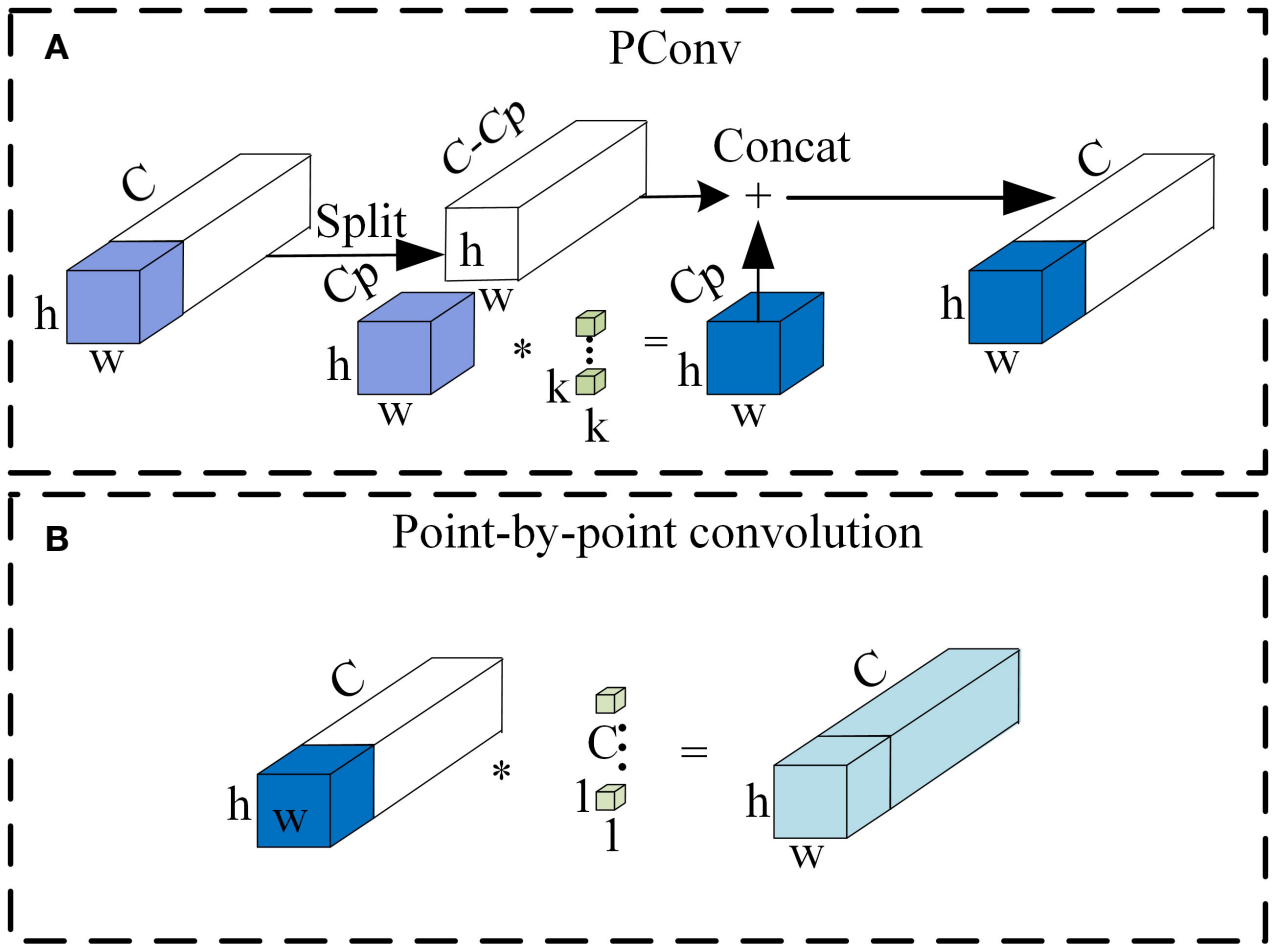
Schematic diagram of the partial convolution structure **(A)** PConv. **(B)** Point-by-point convolution.

Partial convolution (PConv), introduced by Chen et al. (2023), replaces traditional convolution in neural networks with a lightweight alternative. Its principle aims to address the high similarity issue between channels in standard convolution layers. PConv selectively uses a subset of channels for feature extraction, rather than all channels. Extracted features are concatenated with remaining channels, followed by pointwise convolution to enhance channel correlation. Adopting this method significantly reduces computational demands and weight bias, improving classification accuracy. **Figure 4** illustrates the operational process of PConv.

#### 3.2.2 Partial mixed depth separable convolution

**Figure 5:**
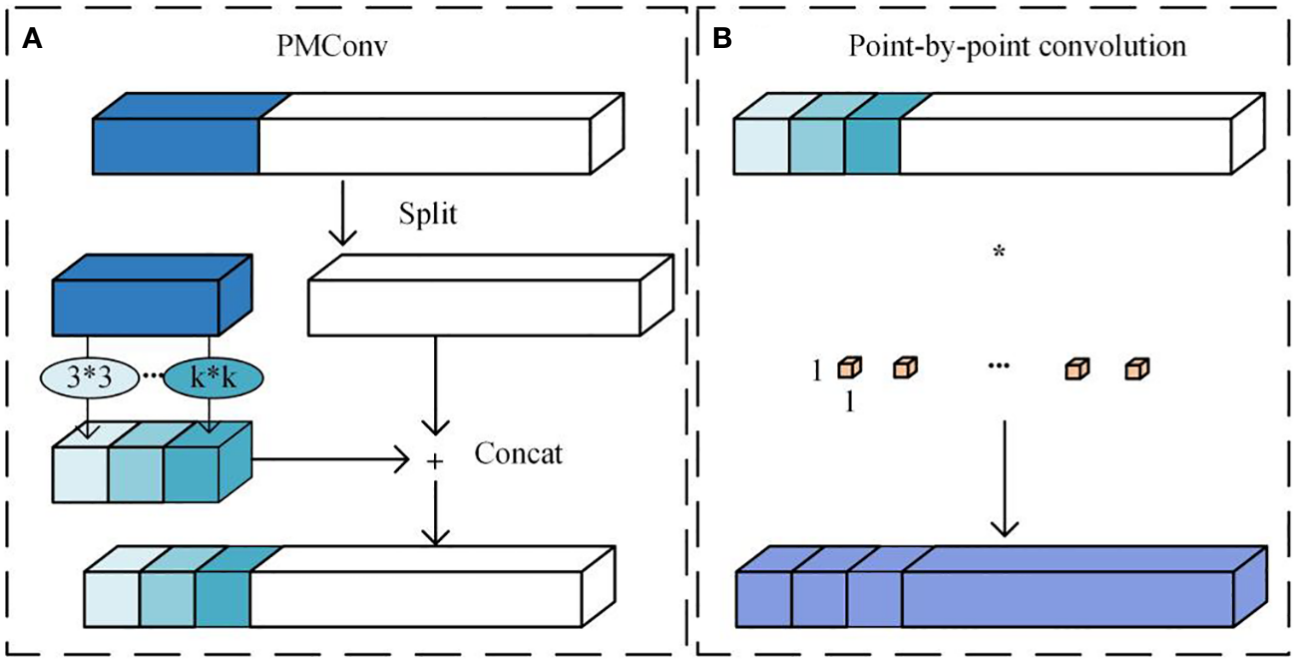
Partial mixed depth separable convolution **(A)** PMConv. **(B)** Point-by-point convolution.

Partial mixed depthwise separable convolution (PMConv) integrates the strengths of Partial convolution (PConv) and Mixed depthwise separable convolution (MixConv), as developed by Tan and Le (2019). **Figure 5A** provides a clear visualization of this integrated architecture. In PMConv’s workflow, an initial screening selects a subset of channels for convolution with various kernel sizes to generate feature maps. Subsequently, pointwise convolution concatenates these feature maps with unused channels, enhancing information exchange and inter-channel correlation. This design enhances the model’s representational capabilities and overall performance.

PMConv reduces computational complexity by excluding redundant features, preventing weight shifts caused by duplicate feature values. It also integrates MixConv, allowing for the integration of feature mappings from diverse receptive fields. **Figure 5** schematically depicts this process. In this approach, PMConv selectively processes a subset of channels, using diverse-sized kernels for convolution to generate feature maps. Subsequent pointwise convolution strengthens the correlation between generated feature maps and unused channels. This approach enables PMConv to combine the advantages of Partial Convolution—significantly reducing computational demands and avoiding weight biases—and the strengths of Mixed Depthwise Separable Convolution to effectively merge features from different receptive fields.

#### 3.2.3 Efficient channel attention mechanism

ECANet (Wang et al., 2020) enhances SENet (Hu et al., 2018). Analysis and evaluation reveal that dimensionality reduction hampers channel attention prediction and that capturing dependencies across all channels is inefficient and superfluous. Conversely, suitable cross-channel interactions can simplify the model without significantly compromising performance.

**Figure 6:**
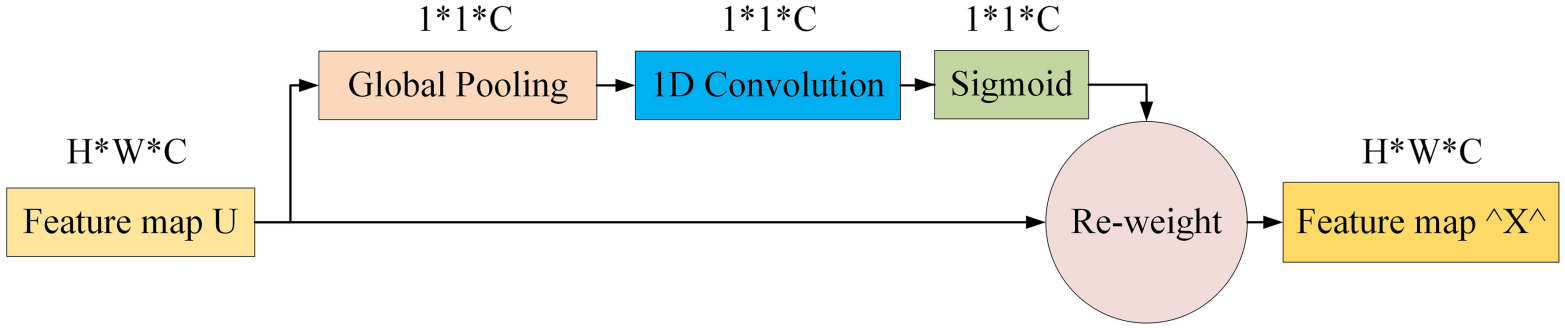
Diagram of efficient channel attention module.

ECANet retains channel dimensionality after global average pooling, obviating reduction. Moreover, its architecture enables local interactions by considering each channel and its adjacent k channels. This approach boosts efficiency in managing channel relationships, enhancing performance. **Figure 6** clearly illustrates ECANet’s structure, showcasing its innovations and advantages. These refinements allow ECANet to overcome SENet’s limitations, offering a streamlined, effective solution for channel attention prediction.

ECANet and SENet differ in several key aspects. Firstly, the conventional MLP module, comprising a fully connected layer (FC1), ReLU activation function, and another fully connected layer (FC2), undergoes an innovative transformation in ECANet, where a one-dimensional convolutional form replaces it. This shift reduces computational burden and enhances efficiency by simplifying parameter calculations. Secondly, the one-dimensional convolution interacts with a subset of channels, streamlining the computation process. This design enables effective cross-channel interactions, maintaining performance while reducing complexity. Lastly, ECANet features an adaptive mechanism that dynamically adjusts the one-dimensional convolution kernel size (k), determining interaction coverage. The value of k proportionally scales with channel dimensions, defined by a specific formula. This strategy endows ECANet with flexibility to adapt to varied data characteristics and demands, enhancing performance and generalization. It is calculated as shown in Equation 1:


(1) $$k={\left|\frac{{\text{log}}_{2}C+\text{b}}{\text{γ}}\right|}_{\text{odd}}$$

Where: C indicates the number of channels, ||_odd_ means that k can only take odd numbers, γ and b are set to 2 and 1 in the paper and are used to vary the ratio between the number of channels C and the size of the convolutional kernel sum.

#### 3.2.4 Convolutional layer replacing fully connected layer

Pointwise convolution, defined by a 1×1 kernel size, offers several advantages. It functions similarly to a fully connected layer, processing feature maps globally. Additionally, pointwise convolution increases the model’s nonlinearity, thereby enhancing its expressive capabilities. Considering these advantages, this study utilizes pointwise convolution layers as replacements for fully connected layers.

**Figure 7:**
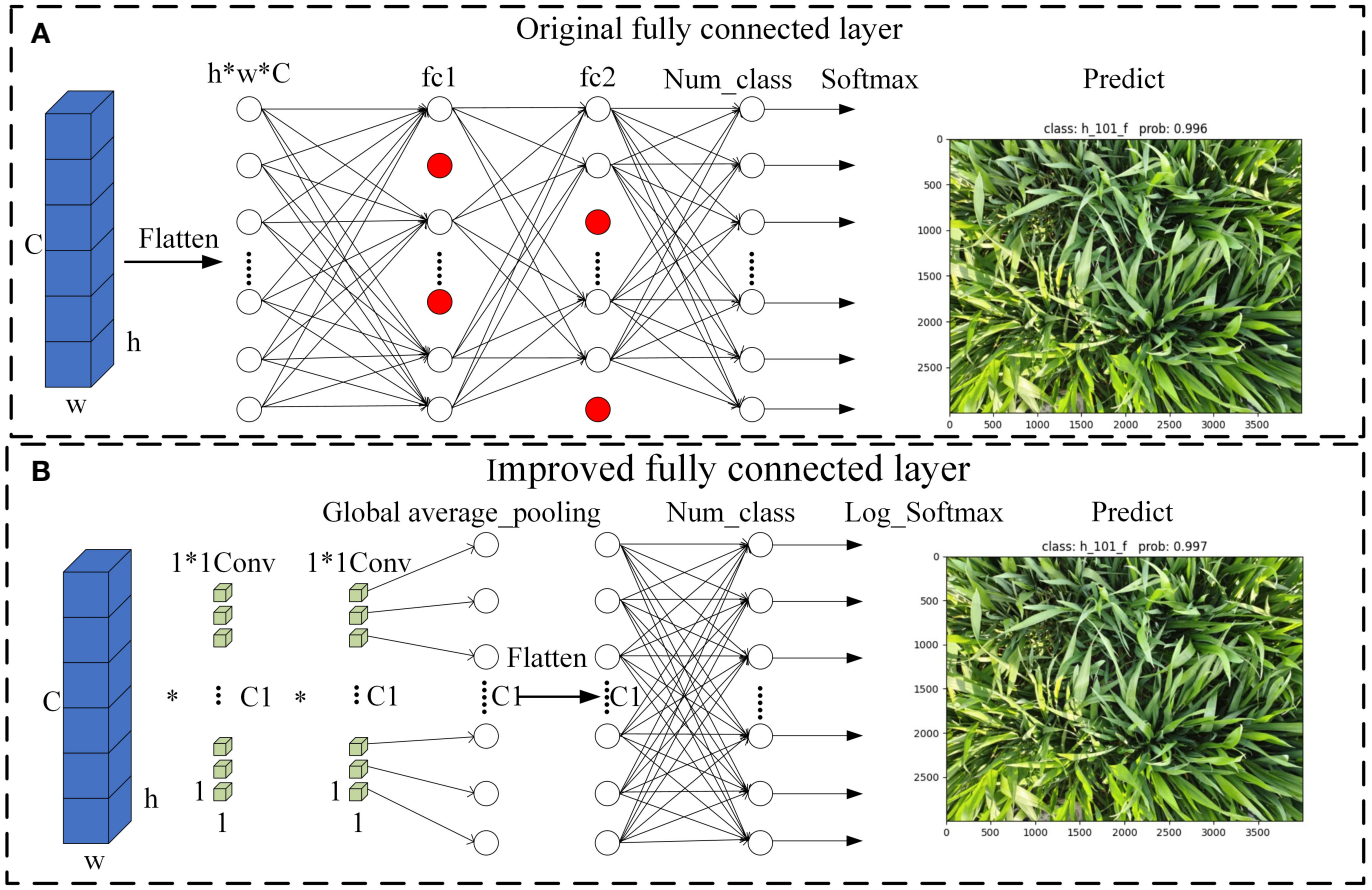
Schematic diagram of the full connectivity layer improvement **(A)** Original fully connected layer. **(B)** Improved fully connected layer.

In the RE-VGG11 model, fully connected layers constitute 92.02% of the total parameters. To optimize model structure, this study replaces the original fully connected layers with two pointwise convolutions and a global average pooling layer. This refinement simplifies the architecture and potentially improves model performance. **Figure 7** illustrates the modifications to the fully connected layers, providing a visual representation of the enhancement.

### 3.3 Parameter setting

#### 3.3.1 Test environment

This study conducted model improvement testing on a laptop using Python in the PyCharm integrated development environment (IDE). **Table 3** meticulously details the laptop’s hardware specifications and PyCharm’s relevant software settings.

**Table 3:** Test equipment parameters.

Configuration	Version
Computer	Legion Y9000P
CPU	Intel Core i7-12700H (3.5GHz/L3 24M)
GPU	NVIDIA GeForce RTX 3070
Memory	8 G
Memory stick	32 G
CUDA	11.6
Cudnn	11.6
Pytorch	1.13.0
Torch vision	0.14.0
Python	3.9.7

#### 3.3.2 Hyperparameter setting

During the training phase, this study utilized the AdamW optimizer (Loshchilov and Hutter, 2018) instead of the original Adam optimizer, significantly improving network model performance.

**Table 4** presents the detailed parameter settings of the network model, carefully adjusted during training to optimize performance.

**Table 4:** Model parameter configuration.

Configuration	Parameters
Optimizer	AdamW (Loshchilov and Hutter, 2018)
Loss function	CrossEntropy loss (Wang et al., 2019)
Batch_size	112
Epoch	120
Initialization rate	0.005
Learning rate decay index	0.5

### 3.4 Evaluation metrics

This study uses a rigorous assessment framework to evaluate the improved model’s performance, covering key metrics such as average accuracy (ACC), F1-score, precision, computational complexity (Flops), memory usage, and runtime. These multi-dimensional evaluation parameters allow for an accurate quantification of the model’s performance, ensuring the experimental results’ reliability and validity. Notably, all mentioned evaluation metrics depend on the confusion matrix for detailed calculations. For matrix parameters involved in training, refer to **Supplementary Table 1**.

(1) Average accuracy rate (ACC).

The average accuracy rate (ACC) is a quantitative metric assessing the model’s prediction accuracy, defined as the ratio of correctly predicted samples to the total predicted samples. It is calculated as shown in Equation 2


(2) $$\text{ACC = }\frac{TP+TN}{TP+FP+TN+FN}$$

(2) Precision and Recall.

Precision and recall are essential for evaluating classifier performance in predicting positive instances, focusing on accuracy and completeness, respectively. Precision represents the ratio of correctly identified positive instances to those classified as positive by the classifier. High precision indicates the classifier’s effectiveness in reducing false positives (FP) by minimizing misclassification of negative instances as positive. It is calculated as shown in Equation 3.


(3) $$\text{Precision(P) = }\frac{TP}{TP+FP}$$

Recall measures the ratio of actual positive instances correctly identified by the classifier. High recall indicates the classifier effectively reduces misclassifications of positive instances as negative, thus lowering the rate of false negatives (FN). It is calculated as shown in Equation 4


(4) $$\text{Recall (R) = }\frac{TP}{TP+FN}$$

(3) F1-score.

The F1-score, incorporating both precision and recall, evaluates the accuracy of model classification. An elevated F1-score signifies enhanced classification performance. It is calculated as shown in Equation 5.


(5) $$F1\_score =\frac{2\times P\times R}{P+R}$$

(4) Flops (Floating point operations).

Flops serve as a metric for quantifying an algorithm or model’s computational complexity, offering insights into resource requirements and efficiency.

(5) Model Memory Occupancy (MB).

This study aims to minimize the model’s parameter count while maximizing accuracy. The model’s memory footprint is a key metric for assessing its superiority in this research context.

(6) Inference speed.

The model’s inference speed is assessed by the total time it takes to process 6,381 testing images. It is calculated as shown in Equation 6:


(6) $$\text{Inference speed = }\frac{x-x1}{x}$$

where x denotes the total inference time spent by the original model and x1 denotes the total inference time spent by the improved model.

## 4 Results and analysis

### 4.1 Comparison of classification modeling results

**Figure 8:**
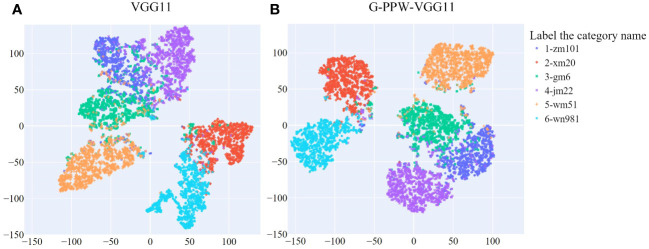
t-SNE visualization of prediction results from different models **(A)** VGG11. **(B)** G-PPW-VGG11.

The original VGG11 and optimized G-PPW-VGG11 models were tested on a dataset of 6,381 testing images. To visually represent prediction outcomes, we used t-SNE (t-Distributed Stochastic Neighbor Embedding) for analysis before and after optimizing the VGG11 model. Results are detailed in **Figure 8**. t-SNE, a two-dimensional dimensionality reduction technique, effectively preserves the local characteristics of high-dimensional data. This ensures data points close in high-dimensional space remain close in the two-dimensional representation. Consequently, the t-SNE visualization in **Figure 8** clearly depicts relationships between different wheat varieties.

**Figure 8** illustrates the relationships among six wheat varieties, showing inter- and intra-class distances and misclassifications. Specifically, Xinmai20, Wanmai51, and Wunong981 show distinct inter-class separations from the other three varieties. Conversely, Zhengmai101 is positioned close to Gaomai6 and JiMai22, indicating less discernible inter-class boundaries.

A comprehensive comparative analysis between the enhanced G-PPW-VGG11 and the original VGG11 models revealed key disparities: (1) A notable increase in the inter-class distance between Jimai22 and Gaomai6 indicates a significant improvement in distinguishing between these varieties. (2) Despite some classification errors among Zhengmai101, Gaomai6, and Jimai22, the model significantly increased the inter-class distances between these varieties. Additionally, the intra-class sample distribution for each variety has become more coherent, demonstrating stronger internal cohesion and reducing intra-class errors. (3) The enhanced model shows a more prominent inter-class distance between Wanmai51 and Gaomai6, with a notable reduction in misclassification events. This underscores the model’s effectiveness in enhancing the discrimination capability between these two varieties.

In conclusion, the findings demonstrate the G-PPW-VGG11 model’s significant advantages in enhancing inter-class distances and minimizing intra-class dispersions. These improvements have enabled the model to achieve superior discrimination between wheat varieties, providing a more accurate and reliable classification approach.

### 4.2 G-PPW-VGG11 ablation test performance comparison

This study performed systematic ablation tests on a dataset comprising 6,381 testing images to assess the efficacy of different optimization strategies. **Table 5** presents the comprehensive results of the ablation experiments.

**Table 5:** Comparison of model improvement results.

Model	Choice						Result				
	a	b	c	d	ACC (%)	F1-score (%)	Precision (%)	Params (MB)	Inference time (s)	Flops (MB)	AUC (%)
VGG11					87.63	87.52	87.69	491.29	144.76	7,618.57	98.46
RE-VGG11					91.76	91.78	91.96	57.63	140.49	878.58	99.27
1	√				92.69	92.65	92.64	50.30	91.35	433.23	99.36
2	√	√			92.75	92.74	92.75	1.78	86.68	426.87	99.35
3	√	√	√		93.00	92.94	92.91	1.79	88.23	426.87	99.41
4	√	√	√	√	**93.52**	**93.47**	**93.47**	**1.79**	**93.45**	**426.87**	**99.32**

(a) PConv. (b) FL2PWConv. (c) PMConv. (d) ECANet.

The bolded data represent the results of the final improved model proposed in this paper.

**Table 5** data indicates Scheme 4’s superior performance, leading to its selection as the final optimized model, G-PPW-VGG11, for this study. The G-PPW-VGG11 model achieved a classification accuracy of 93.52% and a processing time of 93.45s. Notably, the model demonstrated exceptional memory efficiency, using only 1.79 MB. Compared to RE-VGG11, G-PPW-VGG11 improved classification accuracy by 1.76%, speed by 33.48%, AUC value by 0.05%, and reduced memory usage by 96.89%. Additionally, computational complexity decreased by 51.41%. These results indicate that G-PPW-VGG11 significantly optimized resource utilization efficiency while maintaining high classification performance.

Introducing PConv boosted classification speed by 34.98% and reduced computational complexity by 50.69% (445.35 M). FL2PWConv significantly optimized memory, reducing usage by 96.91% (55.85 MB). Incorporating PMConv slightly increased memory use by 0.01 MB but improved classification accuracy by 0.25%. The integration of ECANet improved classification accuracy by 0.52%, without increasing memory usage or computational complexity.

These findings highlight G-PPW-VGG11’s successful balance between classification accuracy and inference speed. The optimized model, G-PPW-VGG11, marks a significant advancement, offering a solution for memory-constrained environments without compromising performance.

**Figure 9:**
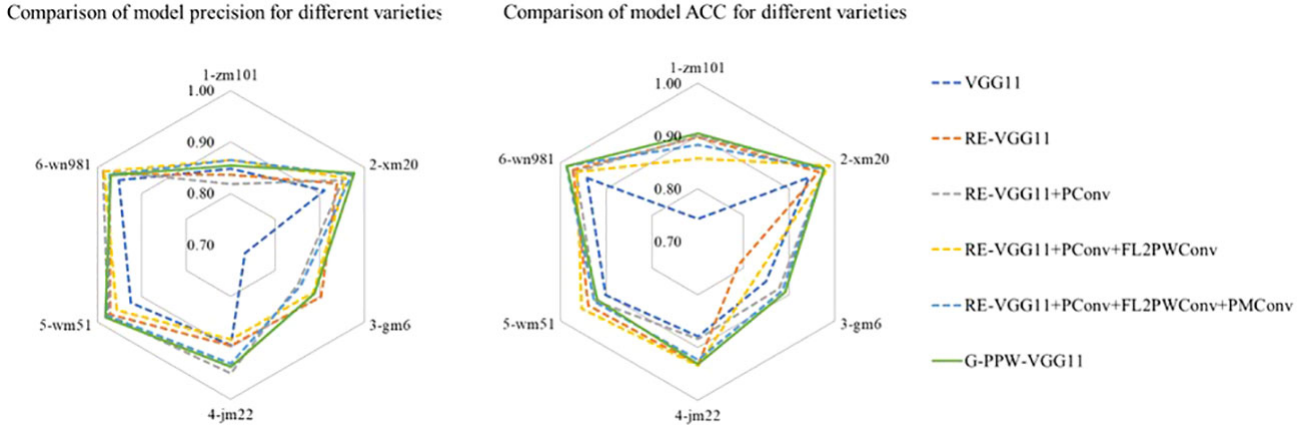
Comparison chart of ablation test results.

**Figure 9** shows a comparative analysis of classification accuracy and precision for six wheat varieties using the optimized models listed in **Table 5**. The results indicate that introducing PMConv enhances classification accuracy for the Zhengmai101 variety without affecting overall accuracy. Furthermore, the evolution into G-PPW-VGG11 results in significant improvements in classification precision and overall accuracy for all wheat varieties. These findings underscore the proposed model’s efficacy in wheat variety classification, particularly highlighting G-PPW-VGG11’s enhanced classification performance.

**Figure 10:**
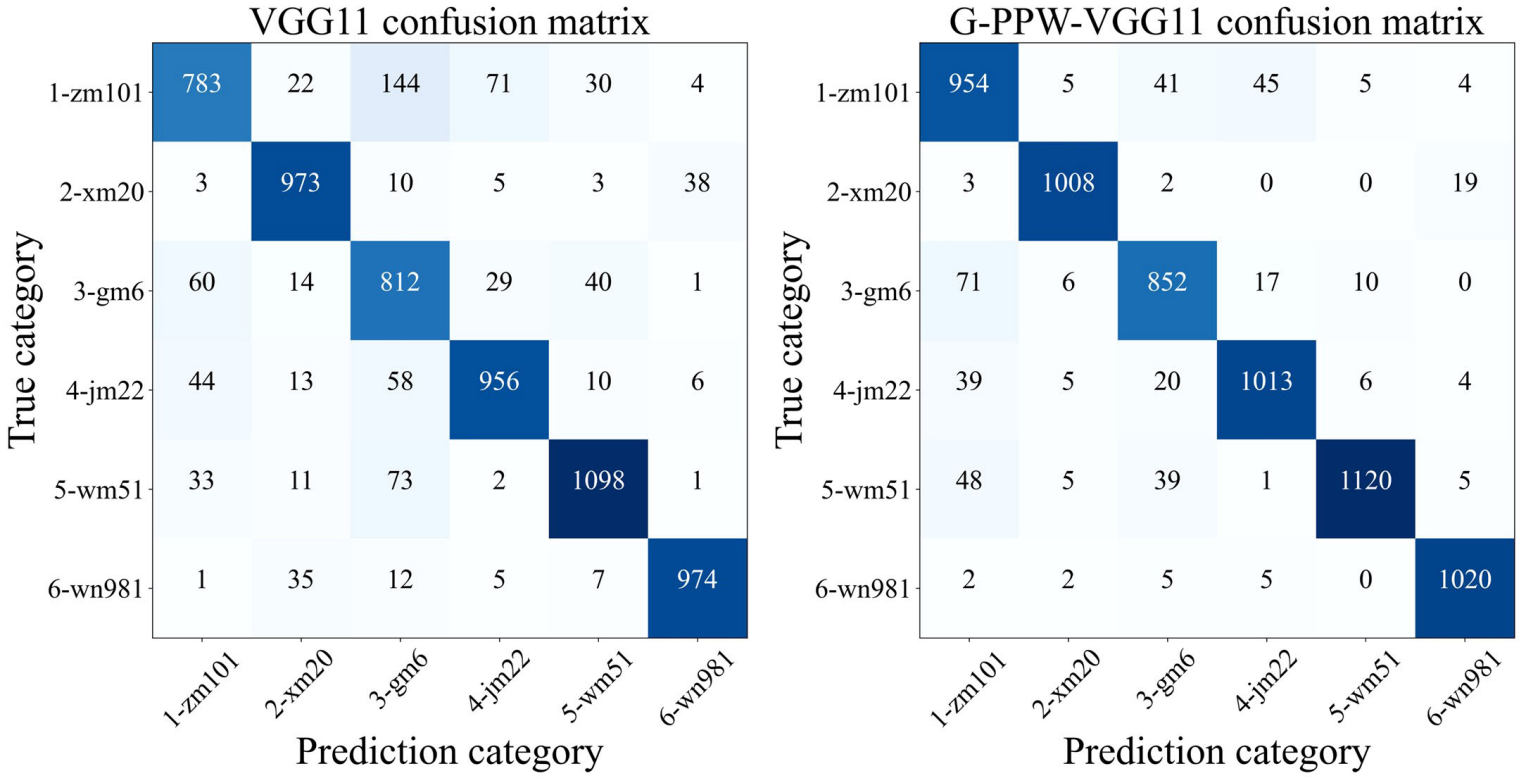
Confusion matrix for VGG11 and G-PPW-VGG11.

An in-depth comparative analysis of confusion matrices, as depicted in **Figure 10**, showed varied improvements in classification accuracy across all six wheat varieties. Specifically, Zhengmai101’s classification accuracy improved by 16.22%, underscoring the substantial enhancement from model optimization. In contrast, Wanmai51’s classification accuracy improved by a modest 1.8%. Nevertheless, this underscores the model improvement’s positive impact on overall classification performance.

Furthermore, the analysis shows a significant reduction in the probability of Zhengmai101 being misclassified as Gaomai6 post-optimization, crucial for minimizing misidentification. Remarkably, post-optimization, the misclassification of Xinmai20 as Jimai22 and Wanmai51 dropped to zero, affirming the optimized model’s efficacy in improving accuracy and reducing misclassifications.

In conclusion, the improved model exhibits superior classification performance for wheat varieties by enhancing accuracy and significantly reducing misclassification risks. This results in a more accurate and reliable tool for wheat variety identification.

**Figure 11:**
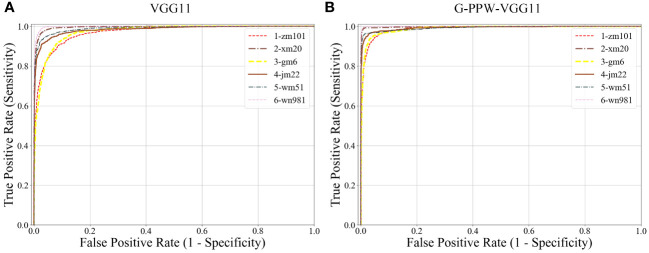
Comparison of ROC curve results **(A)** VGG11. **(B)** G-PPW-VGG11.

**Figure 11** presents a comparative analysis of ROC curves for various wheat varieties, before and after model improvement, using confusion matrix data. The results show that the enhanced model, G-PPW-VGG11, has ROC curves closer to the point (0,1) across all varieties, with higher AUC values. This trend signifies a substantial improvement in the AUC metric for the refined model, indicating superior classification performance. It can be concluded that modifications have significantly enhanced the G-PPW-VGG11 model’s classification accuracy, demonstrating improved discriminatory power and practical value. This finding highlights the critical role of model optimization in improving the accuracy and reliability of wheat variety classification.

### 4.3 Experimental comparison of different lightweight classification models

This study introduces G-PPW-VGG11, a performance-optimized deep learning model designed for wheat canopy leaf image cultivar classification. To validate this model’s effectiveness and superiority, a systematic comparative analysis was conducted against prevalent lightweight network architectures. Specifically, the comparison included benchmark models such as FasterNet, MobileNet_V2, MobileNet_V3 (large and small variants), ShuffleNet_V2 (x1_0 and x1_5 configurations), and EfficientNet’s baseline and sub-baseline models, b0 and b1. The comprehensive results of these comparative experiments are documented in **Table 6**. Meticulous analysis of the experimental data unequivocally demonstrated G-PPW-VGG11’s exceptional performance in wheat canopy leaf image classification, highlighting its potential advantages and application prospects compared to leading lightweight models.

**Table 6:** Comparison of experimental performance of different network models.

Model	ACC (%)	F1-score (%)	Precision (%)	Params (MB)	Inference time (s)	Flops (M)	AUC (%)
FasterNet	89.19	89.16	89.34	52.30	122.52	1910	98.84
MobilNet_V2	89.96	89.94	90.03	8.51	132.04	318.96	98.99
MobilNet_V3_large	88.32	88.23	88.35	16.06	143.43	226.44	98.59
MobilNet_V3_small	87.78	87.79	88.04	5.81	133.58	58.79	98.45
ShuffleNet_V2_x1_0	87.85	87.81	87.84	4.81	138.28	149.58	98.51
ShuffleNet_V2_x1_5	88.69	88.72	88.08	9.48	146.84	302.65	98.63
EfficientNet_b0	92.61	92.56	92.52	15.32	167.79	398.03	99.33
EfficientNet_b1	92.36	92.34	92.33	24.88	210.70	587.07	99.31
G-PPW-VGG11	**93.52**	**93.47**	**93.47**	**1.79**	**93.45**	**426.87**	**99.32**

The bolded data represent the results of the final improved model proposed in this paper.

The results (**Table 6**) reveal that the G-PPW-VGG11 model achieved a classification accuracy of 93.52% and an inference time of 93.45s. Compared to state-of-the-art models like FasterNet, MobileNet_V2, MobileNet_V3_large, MobileNet_V3_small, ShuffleNet_V2_x1_0, ShuffleNet_V2_x1_5, EfficientNet_b0, and EfficientNet_b1, the G-PPW-VGG11 showed superior performance. Specifically, it outperformed these models in terms of classification accuracy by 4.33%, 3.56%, 5.20%, 5.74%, 5.67%, 4.83%, 0.91%, and 1.16%, respectively. Moreover, the model reduced inference time by 29.07s, 38.59s, 49.98s, 40.13s, 44.83s, 53.39s, 74.34s, and 117.25s compared to the aforementioned models.

Notably, the G-PPW-VGG11 model achieved the highest classification accuracy with minimal inference time. Additionally, its 1.79 MB memory footprint makes it highly suitable for mobile deployment, facilitating portable wheat variety classification. These characteristics make the G-PPW-VGG11 model a promising candidate for real-world applications prioritizing accuracy, efficiency, and portability.

**Figure 12:**
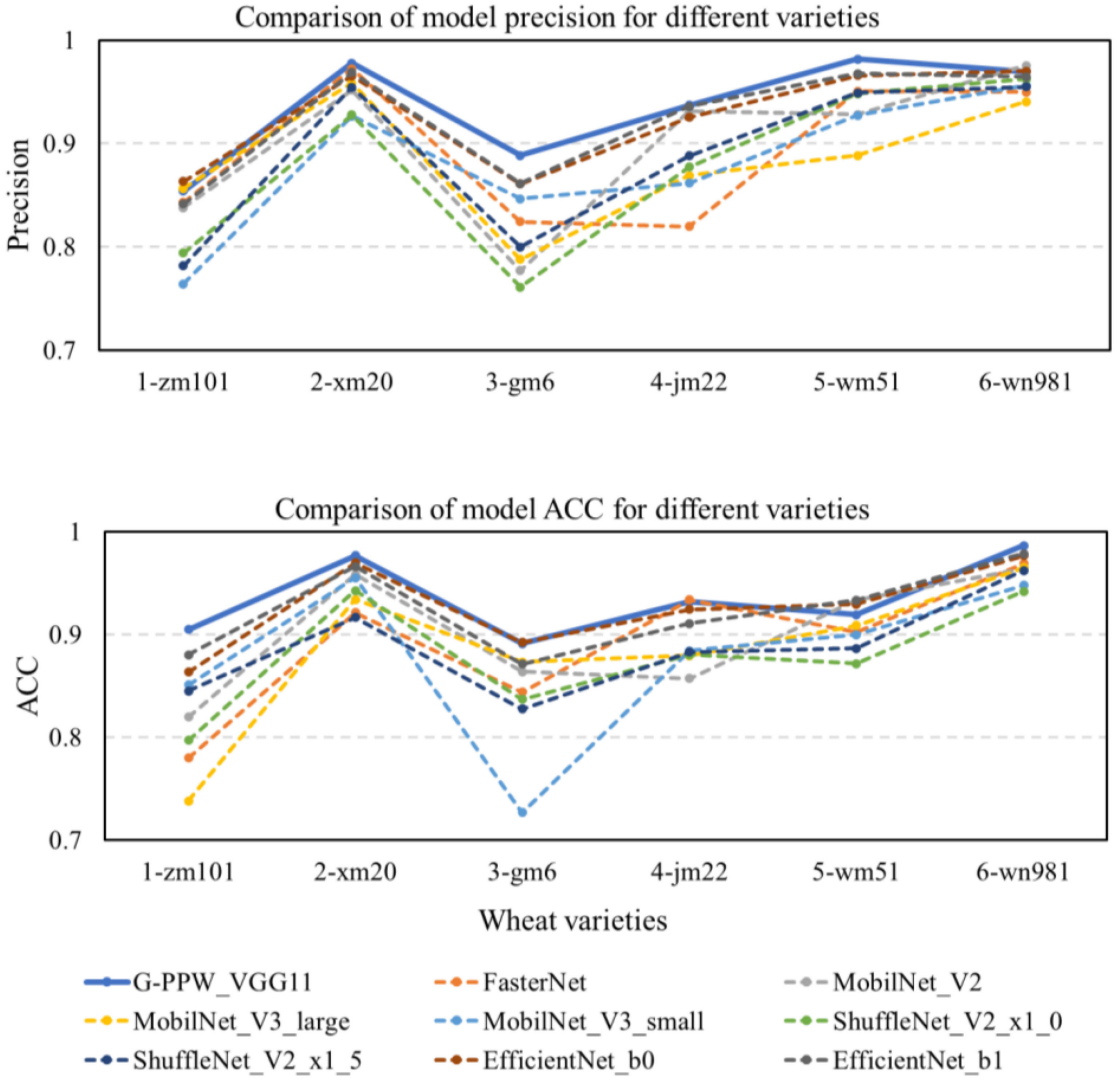
Comparison of model prediction results for each variety.

**Figure 12** presents a comparative diagram of prediction results from various models. Rigorous analysis reveals that the optimized G-PPW-VGG11 model exhibits notable superiority in precision and accuracy (ACC). Among the models compared, G-PPW-VGG11 achieves the highest precision and accuracy, demonstrating its exceptional performance in wheat varieties classification.

### 4.4 Research on model stability testing based on cross-validation

To assess the model’s stability, 3-fold cross-validation was performed on the dataset shown in **Table 2**. The experimental results are detailed in **Table 8**.

The results (**Table 7**) show that the model attained an average classification accuracy of 92.78% following a 3-fold cross-validation process. Compared to the results from the dataset in **Table 1**, there was a minor decrease in classification accuracy, a reduction of 0.74%. This observation suggests that dataset selection influences the model’s classification accuracy. However, the overall impact was not significant, demonstrating the improved model’s robust stability.

**Table 7:** 3-fold cross-validation results.

	Training	Testing	ACC (%)	F1-score (%)	Inference time (s)	AUC (%)
1	Datasets 1 and 2	Dataset 3	93.34	93.32	92.22	99.27
2	Datasets 1 and 2	Dataset 2	92.72	92.75	89.32	99.12
3	Datasets 1 and 2	Dataset 1	92.29	92.30	89.03	98.71

### 4.5 APP result demo

The combined experimental results from Sections 4.3 and 4.4 indicate that the G-PPW-VGG11 model exhibits outstanding classification performance. Specifically, the model achieved an average classification accuracy of 93.52% with a compact size of 1.79MB, making it suitable for use in mobile devices for portable applications. The model was successfully transferred to a Xiaomi 10s Android smartphone using a network/data cable. Practical runtime tests on the device showed a memory usage of 88.50 MB, indicating efficient performance on mobile platforms.

To validate the APP’s practical effectiveness in wheat variety classification, a test dataset of 300 wheat canopy leaf images was collected under field conditions using a smartphone. Rigorous testing and analysis yielded the experimental results presented in **Table 8**. These findings offer valuable insights for future research and contribute to advancing agricultural intelligence.

**Table 8:** Results of the APP identification results.

Variety	Sample number	Correctly identify	ACC (%)	Speed (ms/per image)
Zhengmai101 (zm101)	50	39	78%	286.62
Xinmai20 (xm20)	50	35	70%	293.3
Gaomai6 (gm6)	50	45	90%	277.76
Jimai22 (jm22)	50	48	96%	302.53
Wanmai51 (wm51)	50	38	76%	298.66
Wunong981 (wn981)	50	49	98%	287.38

The results (**Table 8**) show that testing large images (4,000×3,000 pixels) with the APP resulted in an average classification accuracy of 84.67% and an average inference time of 291.04ms. This performance suggests the model meets real-time classification requirements for wheat varieties in field environments, highlighting its practicality and application value.

**Supplementary Figure 2** demonstrates the APP’s ability to read and identify images from the photo gallery. Upon image selection, the APP interface displays the chosen image, predicted variety, inference time, variety characteristics, and cultivation site information. The example shows the predicted wheat variety as Gaomai6, with an inference time of 280ms, confirming the model’s efficiency and accuracy in practical applications.

## 5 Conclusion

This study presents an innovative lightweight convolution method, partial mixed depthwise separable convolution (PMConv), integrating the principles of partial and mixed depthwise separable convolution. Rigorous ablation experiments reveal PMConv’s significant advantages in improving model classification performance.

Employing VGG11 as the baseline architecture, this study integrates PConv with PMConv techniques to accurately capture wheat leaf features. This design reduces misclassification due to weight bias and enhances the model’s feature fusion capabilities. Additionally, the study replaces traditional fully connected layers with FL2PWConv, enhancing nonlinear expressiveness and reducing parameter size for model lightweight. Integrating ECANet allows the model to focus more precisely on critical wheat leaf features, effectively filtering out background noise and significantly improving classification accuracy.

The G-PPW-VGG11 model, as improved in this study, demonstrated exceptional performance with a memory footprint of merely 1.79 MB and a classification accuracy of 93.52%. To validate the model’s capabilities comprehensively, an experimental comparison was conducted between G-PPW-VGG11 and classic lightweight models such as FasterNet, MobileNet_v2, MobileNet_v3_large, MobileNet_v3_small, EfficientNet_b0, EfficientNet_b1, ShuffleNet_V2_x1_0, and ShuffleNet_V2_x1_5. Experimental results showed significant improvements in G-PPW-VGG11 across multiple evaluation metrics. Specifically, G-PPW-VGG11 showed notable improvements over the baseline VGG11 in ACC, F1-score, and precision, with increases of 5.89%, 5.93%, and 5.78%, respectively. These enhancements substantiate the improved model’s superiority in classification performance. Additionally, G-PPW-VGG11 showed a 35.44% improvement in inference time, significantly enhancing response speed. Furthermore, the model reduced memory usage by 99.64% compared to the VGG11 model’s 489.5 MB memory footprint.

To improve portability and classification efficiency, the enhanced model was adapted for testing on the Android smartphone platform. Actual measurements showed that the model recognized a single image in an average of 291.04ms, meeting the stringent criteria for real-time classification. In natural environments, the model’s recognition of wheat varieties demonstrated superior performance, offering valuable insights for research and applications in intelligent agriculture.

To facilitate further research, the trained model and complete dataset from this study have been made publicly accessible. The model code is located in https://github.com/mengyuqq/G-PPW-VGG and The dataset is available at the following URL (https://pan.baidu.com/s/107ICGZOxmOXURkZQcHgbeQ) with the access code: 6666. These contributions are anticipated to serve as valuable resources, providing significant data support and a strong foundation for enhancing the accuracy and efficiency of wheat variety classification in the field.

## Data availability statement

The original contributions presented in the study are included in the article/**Supplementary Material**. Further inquiries can be directed to the corresponding authors.

## Author contributions

YP: Investigation, Methodology, Resources, Software, Validation, Visualization, Writing – original draft, Writing – review & editing. XY: Writing – review & editing, Resources, Supervision, Validation. JD: Resources, Writing – review & editing. YZ: Resources, Writing – review & editing. SL: Funding acquisition, Methodology, Project administration, Supervision, Validation, Writing – review & editing. XJ: Methodology, Project administration, Resources, Supervision, Validation, Writing – review & editing.
