# Supplementary material for: Classification of field wheat varieties based on a lightweight G-PPW-VGG11 model
Source: Front Plant Sci. 2024 May 14;15:1375245. doi: 10.3389/fpls.2024.1375245 (PMC11145979; doi:10.3389/fpls.2024.1375245)
Supplement: Supplementary file 1 [file DataSheet_1.pdf]

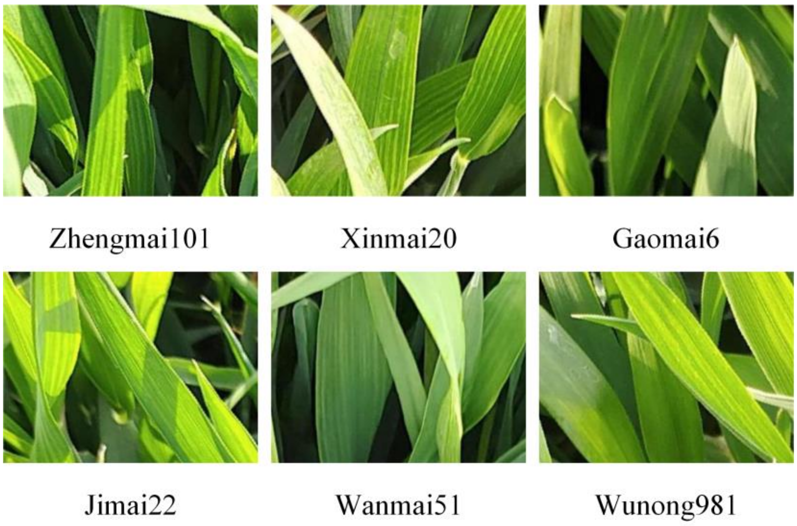

Fig. 1. Images of each species after data preprocessing.

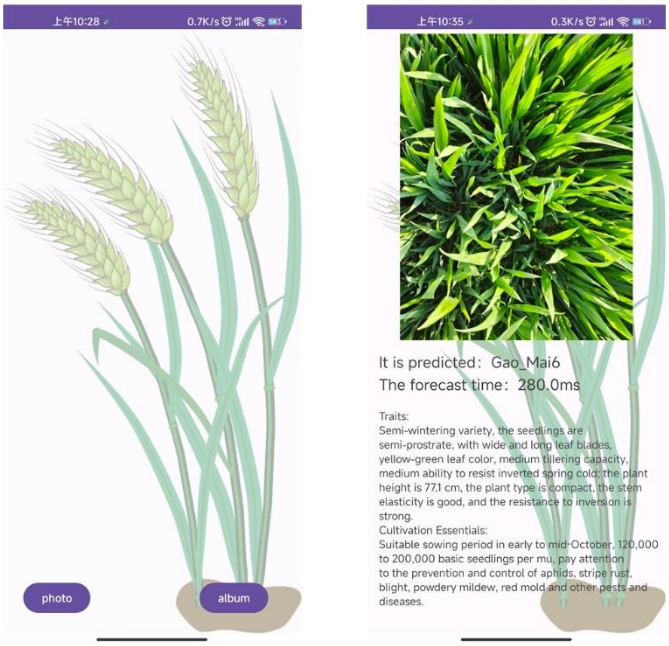

Fig. 2. APP result demo.

Table 1 Confusion matrix.

Confusion matrix		Predicted category	
		Positive	Negative
True category	Positive	True Positive (TP)	False Negative (FN)
	Negative	False Positive (FP)	True Negative (TN)
